# Characterization of Nasco grape pomace-loaded nutriosomes and their neuroprotective effects in the MPTP mouse model of Parkinson’s disease

**DOI:** 10.3389/fphar.2022.935784

**Published:** 2022-08-17

**Authors:** Pathik Parekh, Marcello Serra, Mohamad Allaw, Matteo Perra, Jacopo Marongiu, Giulia Tolle, Annalisa Pinna, Maria Antonietta Casu, Maria Manconi, Pierluigi Caboni, Olivier J. J. Manzoni, Micaela Morelli

**Affiliations:** ^1^ Department of Biomedical Sciences, Section of Neuroscience, University of Cagliari, Cagliari, Italy; ^2^ Department of Life and Environmental Sciences, University of Cagliari, Cagliari, Italy; ^3^ National Research Council of Italy, Institute of Neuroscience, Cagliari, Italy; ^4^ CNR Institute of Translational Pharmacology, Cagliari, Italy; ^5^ INMED, INSERM U1249, Marseille, France; ^6^ Aix-Marseille University, Marseille, France

**Keywords:** grape pomace extract, neurodegeneration, antioxidant activity, neuroprotection, nanotechnology, tyrosine hydoxylase

## Abstract

Grape pomaces have recently received great attention for their richness in polyphenols, compounds known to exert anti-inflammatory and antioxidant effects. These pomaces, however, have low brain bioavailability when administered orally due to their extensive degradation in the gastrointestinal tract. To overcome this problem, Nasco pomace extract was incorporated into a novel nanovesicle system called nutriosomes, composed of phospholipids (S75) and water-soluble maltodextrin (Nutriose^®^ FM06). Nutriosomes were small, homogeneously dispersed, had negative zeta potential, and were biocompatible with intestinal epithelial cells (Caco-2). Nasco pomace extract resulted rich in antioxidant polyphenols (gallic acid, catechin, epicatechin, procyanidin B2, and quercetin). To investigate the neuroprotective effect of Nasco pomace in the subacute 1-methyl-4-phenyl-1,2,3,6-tetrahydropyridine (MPTP) mouse model of Parkinson’s disease (PD), Nasco nutriosomes or Nasco suspension was administered intragastrically and their neuroprotective effects were evaluated. Degeneration of nigro-striatal dopaminergic neurons induced by subacute MPTP treatment, the pathological hallmark of PD, was assessed through immunohistochemical evaluation of tyrosine hydroxylase (TH) in the caudate-putamen (CPu) and substantia nigra pars compacta (SNc), and the dopamine transporter (DAT) in CPu. Immunohistochemical analysis revealed that Nasco nutriosomes significantly prevented the reduction in TH- and DAT-positive fibres in CPu, and the number of TH-positive cells in SNc following subacute MPTP treatment, while Nasco suspension counteracted MPTP toxicity exclusively in SNc. Overall, these results highlight the therapeutic effects of Nasco pomace extract when administered in a nutriosome formulation in the subacute MPTP mouse model of PD and validate the effectiveness of the nutriosome preparation over suspension as an innovative nano-drug delivery system for *in vivo* administration.

## Introduction

Parkinson’s disease (PD) is a neurodegenerative disorder with a multifactorial aetiology and heterogeneous clinical presentation (Obeso et al., 2017). The key neuropathological features of PD include the progressive degeneration of nigro-striatal dopaminergic neurons and the deposition of intraneuronal alpha-synuclein aggregates (Poewe et al., 2017). Despite significant progress being made in the discovery and characterization of genetic and environmental factors contributing to PD (Ascherio and Schwarzschild, 2016; Poewe et al., 2017) at present, there is no preventive or curative intervention (Fenu et al., 2009). Current PD treatments are, in fact, only meant to manage the motor and non-motor symptoms affecting patients.

Neurotoxins, such as 6-hydroxydopamine (6-OHDA), 1-methyl-4-phenyl-1,2,3,6-tetrahydropyridine (MPTP), and rotenone have been widely used in preclinical studies to develop animal models that can recapitulate the main features of PD (Bové and Perier, 2012). Among them, MPTP, a toxin known to induce PD in humans (Langston et al., 1983) through the inhibition of mitochondrial complex I and the formation of free radicals (Meredith and Rademacher, 2011; Zeng et al., 2018), is one of the most utilized animal models as it has broad accessibility and high reproducibility. Today, the MPTP mouse model of PD constitutes the “gold standard” neurotoxin-based animal model to test the effectiveness of putative neuroprotectants (McDowell and Chesselet, 2012).

Several studies have shown that numerous plant extracts and phytochemicals, including polyphenols, flavonoids, and terpenes can exert anti-inflammatory and antioxidant effects in preclinical animal models of neurodegenerative disease, including PD (Kavitha et al., 2020). In particular, *Vitis vinifera* Linn. (family: Vitaceae), a globally widespread grape originating from western Asia and southern Europe (Terral et al., 2010) has received much attention being the richest natural source of antioxidant polyphenols (Suleria et al., 2020). Traditionally, grape extracts have been widely utilized for the treatment of a wide range of health problems including inflammation, cardiovascular disease, hypertension, diabetes, cancer, peptic ulcer, microbial infections, etc. (Gupta et al., 2020). Indeed, several studies have demonstrated the anti-inflammatory, antioxidant, and neuroprotective effects of grape extract *in vitro* and *in vivo* models of brain injury and neurodegenerative diseases (Pazos-Tomas et al., 2020) including Alzheimer’s disease (Wang et al., 2009) and PD, in which the systemic administration of grape seed and skin extract was found to counteract the loss of mesencephalic dopaminergic neurons observed in mice following the unilateral intra-striatal injection of 6-OHDA (Ben Youssef et al., 2021). The process of wine production leads to the generation of a large quantity of grape pomaces, which are generally considered as waste products; however, these pomaces are rich in primary metabolites (i.e., sugars, amino acids, and organic acids) and most importantly in secondary metabolites (i.e., polyphenols) characterized by the presence of relatively varied amounts of phenolic acids (gallic acid), flavanols (catechin, epicatechin, procyanidin B2), and flavonols (quercetin) (Manconi et al., 2020). Interestingly, some of these polyphenols were reported to contrast the loss of mesencephalic dopaminergic neurons observed in the unilateral 6-OHDA-lesioned rodent model of PD (Sriraksa et al., 2012; Teixeira et al., 2013; Bitu Pinto et al., 2015; Zhang et al., 2019). In line with these findings, the cost-effective and sustainable conversion of grape pomaces into valuable polyphenolic extracts, and their subsequent therapeutic uses in multiple neurodegenerative diseases, may represent an innovative and promising strategy through which an environmental burden may be reduced while providing a new source of nutraceuticals.

Some of the major problems associated with the therapeutic use of polyphenols are related to their low bioavailability, especially when administered orally. Therefore, the encapsulation of polyphenol-rich natural extracts is considered to be an effective approach to enhance their bioavailability and penetration into the brain (Grgić et al., 2020; Zhang et al., 2021).

In the present study, we used pomace extracts obtained from the Nasco grape (*Vitis vinifera* L. ssp. vinifera, Vitaceae), a white Italian wine grape variety grown in Sardinia, Italy (Lacombe et al., 2011; Orru et al., 2015). Next, we combined Nasco pomace extract (NPE) with Nutriose^®^ FM06 (Nutriose^®^), a water-soluble branched maltodextrin obtained from maize, and phospholipid S75, to obtain an innovative nano-drug delivery system known as nutriosomes. Thanks to their phospholipid-based nanostructure, nutriosomes enable the oral delivery of both hydrophilic and lipophilic agents and significantly improve their systemic bioavailability by increasing the adherence of the formulation to the intestinal mucosa (Castangia et al., 2015; Catalán-Latorre et al., 2018). Moreover, Nutriose^®^ stabilizes the structure of vesicles (Martí Coma-Cros et al., 2018) and improves the resistance of nutriosomes, and their encapsulated polyphenols, to the acidic environment of the gastrointestinal tract (Allaw et al., 2020). In support of nutriosomes as a highly efficient nano-drug delivery system, previous *in vitro* and *ex vivo* studies have demonstrated their potential therapeutic application in the delivery of lipophilic, and otherwise unstable antioxidant compounds, to counteract harmful oxidative stress (Catalán-Latorre et al., 2018; Allaw et al., 2020; Manconi et al., 2020).

On this basis, the present study aimed to: 1) characterize the polyphenolic composition of NPE; 2) assess the main physicochemical properties (size, zeta potential, polydispersity index, and entrapment efficiency) of empty nutriosomes (EN) and Nasco pomace-loaded nutriosomes (NN); 3) test the biocompatibility and antioxidant potential of NN and Nasco suspension (NS; used as control) towards the human intestinal epithelial cells (Caco-2); and 4) investigate the effectiveness of repeated administration of NN and NS against the neurotoxic effects of MPTP in mice at the level of the dopaminergic nigro-striatal system. Specifically, we employed the subacute MPTP mouse model of PD, in which the number and density of tyrosine hydroxylase (TH)-positive neurons and fibres, forming the dopaminergic nigro-striatal system, are significantly reduced (Costa et al., 2013).

## Materials and methods

### Chemicals and drugs

MPTP was purchased from Toronto Research Chemicals, Canada (D463595) and was dissolved in distilled water (VEH). Grape pomaces from Nasco white wine were provided by a local winery (Cantine Argiolas), harvested in southern Sardinia (Italy) in September-October 2020. Samples were dried and stored under vacuum at -20°C until use. Lipoid S75 (S75) was purchased from Lipoid (Ludwigshafen, Germany). Nutriose^®^ was donated by Roquette (Lestrem cedex, France). Ethanol and all other products were of analytical grade and were purchased from Sigma-Aldrich (Milan, Italy). Reagents and plastics for cell culture were purchased from Life Technologies Europe (Monza, Italy).

### Preparation of extract from Nasco grape pomace

Grape pomace was grinded to obtain a powder with small particles (Azwanida, 2015). The resulting grape pomace powder was kept under a vacuum, at room temperature, and protected from light until further extraction. The extraction was performed using a solid-liquid extraction method (Paunović et al., 2014). Briefly, aliquots of 10 g of ground grape pomaces were suspended in 300 ml of ethanol and, a water mixture (70:30 v/v). The suspension was left under constant stirring at 40°C in the dark for 2 h. The maceration was assisted by two ultrasound cycles of 10 min, one at the beginning and the other after 1 h from the extraction process (Romdhane and Gourdon, 2002; Caldas et al., 2018). The obtained suspension was centrifuged (15 min, 4,000 rpm) to remove the coarse fractions. Ethanol was eliminated from the resulted solutions using a rotavapor (Rotavapor RII, BÜCHI Labortechnik AG, Flawil, Switzerland). The final aqueous solution of the extract was lyophilized using a freeze dryer at −80°C for 48 h. After lyophilization, the extract powder was stored under a vacuum in the dark until used (Maier et al., 2009).

### Liquid chromatography-quadrupole-time of flight-mass spectrometry (LC-QTOF-MS) analysis of the NPE

The NPE was characterized qualitatively and quantitatively by LC-QTOF-MS to determine the presence and concentration of five individual phenolic compounds, namely: gallic acid, (+) catechin, (−) epicatechin, procyanidin B2, and quercetin. Briefly, an Agilent 6,560 series ion mobility LC/Q-TOF (Agilent Technologies, Palo Alto, CA, United States) equipped with electrospray ionization interface was employed. After injection of 8 µl of the sample (methanolic solution of dried NPE), an optimal separation was achieved using the mobile phase consisting of water with 0.1 M formic acid (A) and methanol with 0.1 M formic acid (B) using a kinetex Evo column (5 μm, C18, 100 Å; kinetex, Torrance, CA, United States). Gradient elution mode with a flow rate of 0.4 ml/min was used: 0–15 min from 0 to 100% (B); 15–19 min 100% (B); 19–21 min from 100 to 0% (B); 21–24 min 0% (B). The ESI parameters were as follows: nebulizer (20 psi), drying gas (N_2_) flow (5 L/min), and drying gas temp (325°C). The mass spectrometer was used in negative ion mode with a scanning range from *m/z* 40 to 1700. Analysis was performed on MassHunter qualitative analysis workstation software (version 10.0, Agilent technologies). The concentration of each compound was quantified using calibration curves of HPLC grade standards (Gallic acid, PHL89198; (+) Catechin, 43,412; (−) Epicatechin, 68,097; Procyanidin B2, 0984, Extrasynthese, France; Quercetin, Q4951, Merck, Italy) and was expressed as mg/kg of dried pomace.

### Nutriosomes preparation and characterization

Soy-based phospholipid S75 (120 mg/ml), Nasco grape pomace extract (5 mg/ml or 10 mg/ml), olive oil (100 mg/ml), and Nutriose^®^ (400 mg/ml) were weighed in a glass vial and hydrated with a propylene glycol and water (50:50) mixture (Supplementary Table S1). The obtained dispersion was sonicated (10 and 15 cycles: 5 s on and 2 s off; 13 µm of probe amplitude, allowing cooling between each sonication to prevent excessive heating), using a Soniprep 150 ultrasonic disintegrator (MSE Crowley, London, United Kingdom), to obtain homogeneous systems with small particles referred as nutriosomes. Nutriosomes without extract referred to as EN was also prepared and used as a control. The average diameter, polydispersity index, and zeta potential of EN and NN were determined by light scattering using a Zetasizer Ultra (Malvern Instruments, Worcestershire, United Kingdom) (Supplementary Figure S1). Individual values are reported and described in the supplementary data (Supplementary Table S2).

### Effect of NN and NS on the cell viability and hydrogen peroxide (H_2_O_2_)-induced oxidative damage in human intestinal epithelial cells (Caco-2)

Caco-2 cells were grown as monolayers in 75 cm^2^ flasks, incubated under 100% humidity, and 5% CO_2_ at 37°C. Dulbecco’s Modified Eagle Medium-high glucose, containing L-glutamine, supplemented with 10% fetal bovine serum, 1% penicillin, and streptomycin was used as a culture medium. The cultured cells were further used to assess:

1) The effect of NS and NN on the viability of Caco-2 cells. For this experiment, the cells were seeded at a density of 7.5×10^3^ cells/well into 96-well plates. After 24 h of incubation, the cells were treated with either EN, NS, or NN for 48 h.

2) The protective effect of NS and NN on the H_2_O_2_-induced oxidative damage. The cells were seeded at a density of 5×10^4^ cells/well into 96-well plates for 24 h. Further, the cells were exposed to H_2_O_2_ (14.5 μM)-alone or in association with either EN, NS, or NN for 4 h, followed by a rinse with PBS.

For both the experiments, stock solutions of NS and NN (5 mg/ml) were diluted with cell media in a proportion of 1:1,000, 1:10,000 and, 1:100,000 to obtain the final concentrations of: 5, 0.5, and 0.05 μg/ml, respectively. Similarly, stock solutions of NS and NN (10 mg/ml) were diluted with cell media in a proportion of 1:1,000, 1:10,000 and, 1:100,000 to obtain the final concentrations of: 10, 1, and 0.1 μg/ml, respectively. At the end of both experiments, cell viability was assessed by performing the 3-(4,5-dimethylthiazol-2-yl)-2,5-diphenyl tetrazolium bromide (MTT) assay. Briefly, MTT (100 μL, 0.5 mg/ml, final concentration) was added to each well and incubated at 37°C for 3 h. The formed formazan crystals were dissolved in dimethyl sulfoxide, and the absorbance at 570 nm was measured by using a microplate reader (Synergy 4 Reader, BioTek Instruments, AHSI S. p.A, Bernareggio, Italy) (Allaw et al., 2020). The experiments were repeated at least three times and each sample was analyzed in triplicate. The results are shown as a percentage of cell viability and are normalized to untreated Caco-2 cells (100%).

### Animals

Fifty-eight, 16–19-weeks old adult male C57BL/6J mice (Charles River, Calco, Italy), weighing 23–28 g at the beginning of the experiments were used. Mice were housed in a group of 4 per cage under constant temperature and a 12-h light/dark cycle. Standard laboratory chow and tap water were available *ad libitum*. All experiments were conducted in accordance with the guidelines for animal experimentation of the EU directives (2010/63/EU; L.276; 22/09/2010) and with the guidelines issued by the Organism for Animal Welfare (OPBA) of the University of Cagliari. Experiments were designed to minimize animal discomfort and to reduce the number of animals used.

### *In vivo* experimental plan

**FIGURE 1:**
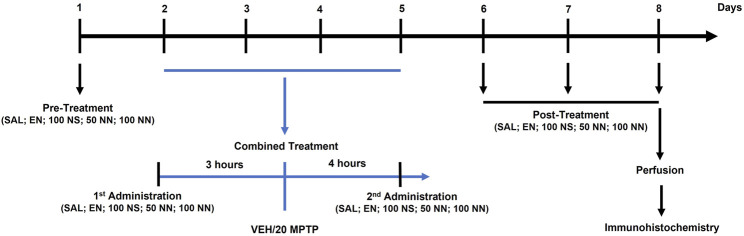
Illustration of the experimental design. Male mice were treated for 8 days as follows. On day 1 (pre-treatment), mice received an intragastric (i.g.) administration of either SAL, EN, NS, or NN. From days 2–5 (combined treatment), mice received once a day: (i) an i.g. administration of either SAL, EN, NS, or NN; (ii) an intraperitoneal (i.p.) injection of either VEH or MPTP (20 mg/kg/day) [3 h after (i)]; and (iii) an i.g. administration of either SAL, EN, NS, or NN [4 h after (ii)]. From days 6–8 (post-treatment), mice received a daily single administration of either SAL, EN, NS, or NN. Lastly, on day 8, 30 min after the last administration, mice were sacrificed for further immunohistochemical analyses. Abbreviations: EN, empty nutriosomes; MPTP, 1-methyl-4-phenyl-1,2,3,6-tetrahydropyridine; NN, Nasco nutriosomes; NS, Nasco suspension; SAL, saline; VEH, distilled water. The numbers indicate the dose of each compound in mg/kg.

Figure 1 depicts the experimental plan:

In experiment 1 (100 mg/kg of NPE), forty-two mice were randomly allocated into five experimental groups and treated with either: (I) Saline + Vehicle: [SAL/VEH, *n* = 12]; (II) Empty nutriosomes (EN)+VEH: [EN/VEH, *n* = 4]; (III) EN + MPTP (20 mg/kg): [EN/MPTP, *n* = 10]; (IV) Nasco suspension (NS, 100 mg/kg)+MPTP (20 mg/kg): [NS (100 mg/kg)/MPTP, *n* = 6]; (V) Nasco nutriosomes (NN, 100 mg/kg)+MPTP (20 mg/kg): [NN (100 mg/kg)/MPTP, *n* = 10].

SAL, EN, NS, and NN were administered intragastrically (i.g.) by oral gavage (18-gauge) in a volume of 10 ml/kg of body weight, while VEH and MPTP (20 mg/kg/day x 4-consecutive days, subacute MPTP-treatment) were given intraperitoneally (i.p.) in a volume of 10 ml/kg of body weight. As depicted in the experimental design (Figure 1), on day 1 (pre-treatment), mice received either SAL, EN, NS, or NN. From day 2 to day 5 (combined treatment), mice received once a day: (A) either SAL, EN, NS, or NN; (B) an i.p. injection of either VEH or MPTP [3 h after (A)]; (C) either SAL, EN, NS, or NN [4 h after (B)]. Lastly, from day 6–8 (post-treatment), mice received a daily single administration of either SAL, EN, NS, or NN.

In experiment 2 (50 mg/kg of NPE), sixteen mice were randomly allocated into four experimental groups and treated with either: (I) Saline + Vehicle: [SAL/VEH, *n* = 4]; (II) EN + VEH: [EN/VEH, *n* = 4]; (III) EN + MPTP (20 mg/kg): [EN/MPTP, *n* = 4]; (IV) NN (50 mg/kg) + MPTP (20 mg/kg): [NN (50 mg/kg)/MPTP, *n* = 4]. The experimental design was the same as experiment 1 (Figure 1).

### Immunohistochemical experiments

#### Tissue preparation

On day 8, thirty minutes after the last administration of either SAL, EN, NS, or NN, mice were deeply anesthetized and transcardially perfused with ice-cold saline (NaCl, 0.9%) followed by 4% paraformaldehyde (PFA) in 0.1 M phosphate buffer (PB, pH 7.4). Subsequently, their brains were isolated, post-fixed in 4% PFA for 2-h, and preserved in PB saline 1X (PBS) at 4°C. The next day, brains were coronally cut on a vibratome to obtain sections (50 μm) suited for immunohistochemical processing.

For each mouse, three coronal sections were obtained according to the following stereotaxic coordinates: A) caudate-putamen (CPu): 1.34 to 0.74 mm, B) substantia nigra pars compacta (SNc): −2.92 to −3.52 mm relative to bregma, according to the mouse brain atlas (Paxinos and Franklin, 2008).

#### TH Immunohistochemistry

Immunohistochemical evaluation of TH was performed in CPu and SNc as described previously (Costa et al., 2014). Briefly, free-floating sections were rinsed three times in PBS 1X and incubated with 1% H_2_O_2_ (30% v/v, Merck) in PBS at room temperature (10 min), to block endogenous peroxidase activity. Then, sections were blocked and permeabilized with 5% normal goat serum and 0.1% Triton X-100 at room temperature (20 min), and later incubated with primary antibody directed towards TH (polyclonal rabbit anti-TH, 1:1,000, AB152, Millipore Corporation, MA, United States) at room temperature (2 nights). Thereafter, sections were incubated with biotinylated secondary antibody (Goat anti-rabbit; 1:500, Vector, Peterborough, United Kingdom), then the avidin–peroxidase protocol (ABC, Vector, Peterborough, United Kingdom) was applied for visualization, using 3,3ʹ-diaminobenzidine (Merck) as a chromogen. Afterward, sections were mounted onto super-frost glass slides, dehydrated, and coverslipped using Eukitt^®^ mounting medium.

#### Immunofluorescence labeling of dopamine Transporter (DAT)

For the immunofluorescence evaluation of DAT in CPu, free-floating sections were rinsed in PB 0.1M, blocked, and permeabilized in 3% normal goat serum and 0.3% Triton X-100 in 0.1 M PB at room temperature (3 h), followed by incubation in the same solution with the primary antibody (monoclonal rat anti-DAT; 1:1,000, MAB369, Millipore, CA, United States) at 4°C (2-nights). Then, sections were rinsed three times in PB 0.1 M and incubated with the biotinylated secondary antibody (biotinylated goat anti-rat; 1:200) at room temperature (2 h), followed by incubation with AlexaFlour^®^ 488-labeled streptavidin (1:500, Jackson ImmunoResearch Europe, Newmarket, United Kingdom) at room temperature (1 h). Afterward, sections were rinsed in PB 0.1 M and mounted onto super-frost glass slides using Mowiol^®^ mounting medium (Costa et al., 2017). Omission of either the primary or secondary antibodies served as negative control and yielded no labeling (data not shown).

#### Image acquisition and analysis of DAT- and TH-positive fibres

Images of a single wavelength were digitalized and captured in RGB by using an epifluorescence microscope (Axio Scope A1, Zeiss, Germany) connected to a digital camera (1.4 MPixels, Infinity 3–1, Lumenera, Canada) as previously described (Costa et al., 2017). Coronal CPu sections were captured at ×10 magnification for the analysis of TH immunoreactivity, or ×20 magnification for the analysis of DAT immunoreactivity. The ImageJ software (National Institutes of Health, United States) was used to quantify the densities of DAT- and TH-positive fibres in CPu. For density quantification, images were first converted to 8-bit, background subtracted, then the densities of immunoreactive fibres were determined by measuring the mean grey density in fixed regions representing the dorsal CPu. Analyses were always performed in a blinded manner in the three sections. No significant differences in the density of DAT- and TH-positive fibres were found among the three sections of a given area in the same mouse. Thus, values from different levels were averaged, normalized with respect to the SAL/VEH group, and expressed as a percentage.

#### Stereological acquisition and analysis of TH-positive neurons in the SNc

Stereological analysis of the total number of TH-positive neurons in the SNc was carried out blind in both hemispheres, using a software (Stereologer) linked to a motorized stage on a light microscope (Casu et al., 2004). The SNc region was outlined at low magnification (×2), according to the atlas of Paxinos and Franklin (Costa et al., 2019), and sampling of cells was achieved by using automatically randomized sampling and optical dissector (50 × 50 × 15 μm). Cells were sampled with a ×40 objective through a defined depth with a guard zone of 2 μm. The coefficient of error ranged from 0.05 to 0.1 (Costa et al., 2019).

### Data analysis and statistics

Statistical analysis was carried out with GraphPad Prism 8 software (GraphPad Software, Inc., La Jolla, CA), and data were analyzed either by One-way or Two-way analysis of variance (ANOVA) followed by either Tukey’s or Newman-Keuls post-hoc test, when applicable. Results are expressed as mean ± SEM and were considered statistically significant if *p* < 0.05.

## Results

### Characterization of polyphenols in NPE

**FIGURE 2:**
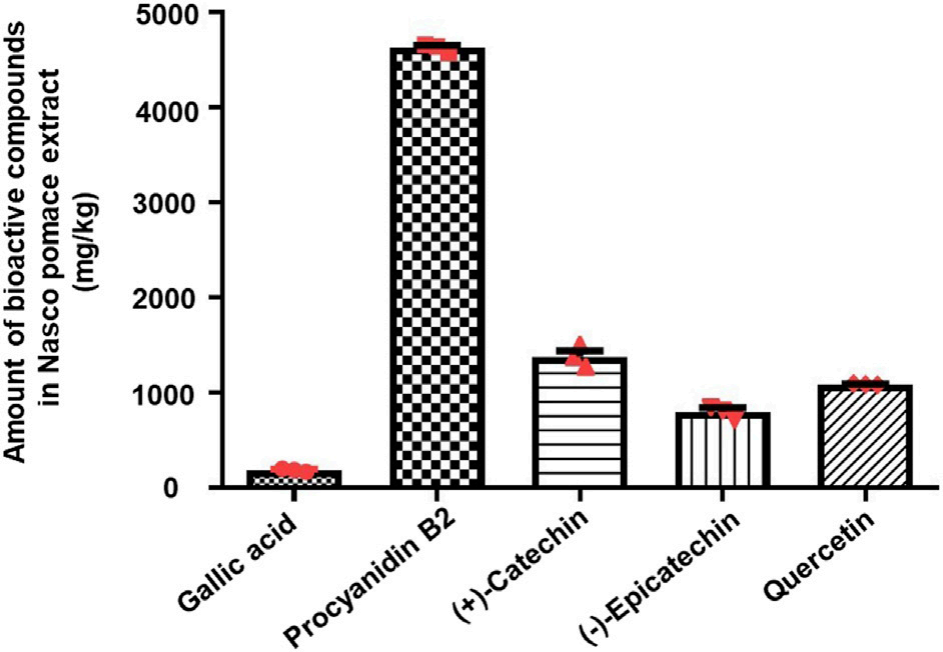
Liquid chromatography–quadruple time-of-flight mass spectrometry quantitative analysis of polyphenols present in NPE. The histogram indicates the amount of bioactive compounds present in NPE (mg/kg). The data are represented as mean ± SEM (*n* = 3). Abbreviations: NPE: Nasco pomace extract.

LC-QTOF-MS analysis indicated the presence of gallic acid, (+)-catechin, (−)-epicatechin, procyanidin B2, and, quercetin **(** Supplementary Figure S2), which was further confirmed by comparing the retention time (RT) of individual compounds from the extract and their relative authentic standards (Supplementary Figure S3). As shown in Supplementary Table S3, the phenolic profile was dominated by the presence of flavonoids (flavan-3-ols and flavonols), while phenolic acid (hydroxybenzoic acid) was found in lower amounts and was characterized by the presence of gallic acid (182 mg/kg). As shown in Figure 2, the monomeric (i.e., (+)-catechin) and oligomeric (i.e., procyanidin B2), flavan-3-ols were the most abundant compounds present in the NPE. Among the monomers, (+)-catechin was the major flavan-3-ol (1,375 mg/kg), while (−)-epicatechin was also present (799 mg/kg). Procyanidin B2 was detected in the highest amount with respect to any other phenolic compounds (4,626 mg/kg). Lastly, quercetin (flavonol) was also detected (1,087 mg/kg).

### Biocompatibility of nutriosomes toward Caco-2 cells

**FIGURE 3:**
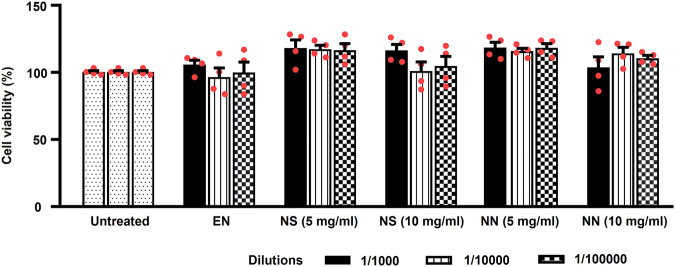
Effect of the NN and NS on the viability of cultured Caco-2 cells. The histogram indicates the viability of Caco-2 cells treated with: either EN, NS (5 or 10 mg/ml), or NN (5 or 10 mg/ml) at three different dilutions (1:1,000, 1:10,000, and 1:100,000). Here, 5 and 10 mg/ml of NN and NS indicate the amount of Nasco pomace loaded inside the nutriosomes and suspension. The data (cell viability, measured by MTT assay) were normalized and expressed as a percentage of the untreated cells (100%). The data are represented as mean ± SEM (*n* = 3). Abbreviations: EN, empty nutriosomes NN, Nasco nutriosomes; NS, Nasco suspension.

Being nutriosomes tailored for intragastric administration and intended to pass the intestinal wall, we evaluated the viability of Caco-2 cells, the most used model of human intestinal epithelial cells (Angelis and Turco, 2011), following 48 h of incubation with either EN, NS, or NN. Herein, EN was used as control, while untreated cells served as reference and were considered to be 100% (Figure 3).

Two-way ANOVA of Caco-2 cells viability showed a main effect of the treatment (F_5,54_ = 7.40, *p* < 0.0001), but not of dilution (F_2,54_ = 0.58, *p* = 0.561), or treatment × dilution interaction (F_10,54_ = 0.87, *p* = 0.565). Incubation with EN did not cause any reduction in cell viability (∼100%) at all the tested dilutions (Figure 3). While, both NS and NN (5 mg/ml) were demonstrated to enhance the cell viability up to 118% at all tested dilutions (Figure 3). Of note, the cell viability was not affected when cells were incubated with higher dilutions (1:10,000 and 1:100,000) of NS (10 mg/ml) (104%). On the contrary, NN (10 mg/ml) was able to maintain the cell viability at ∼110%–114%, even when used at higher dilutions (1:10,000 and 1:100,000) (Figure 3). Importantly, regardless of the concentrations and dilutions of EN, NN, and NS used, they were found to have no toxic effects on Caco-2 cells.

### NN and NS on H_2_O_2_-induced oxidative stress in Caco-2 cells

**FIGURE 4:**
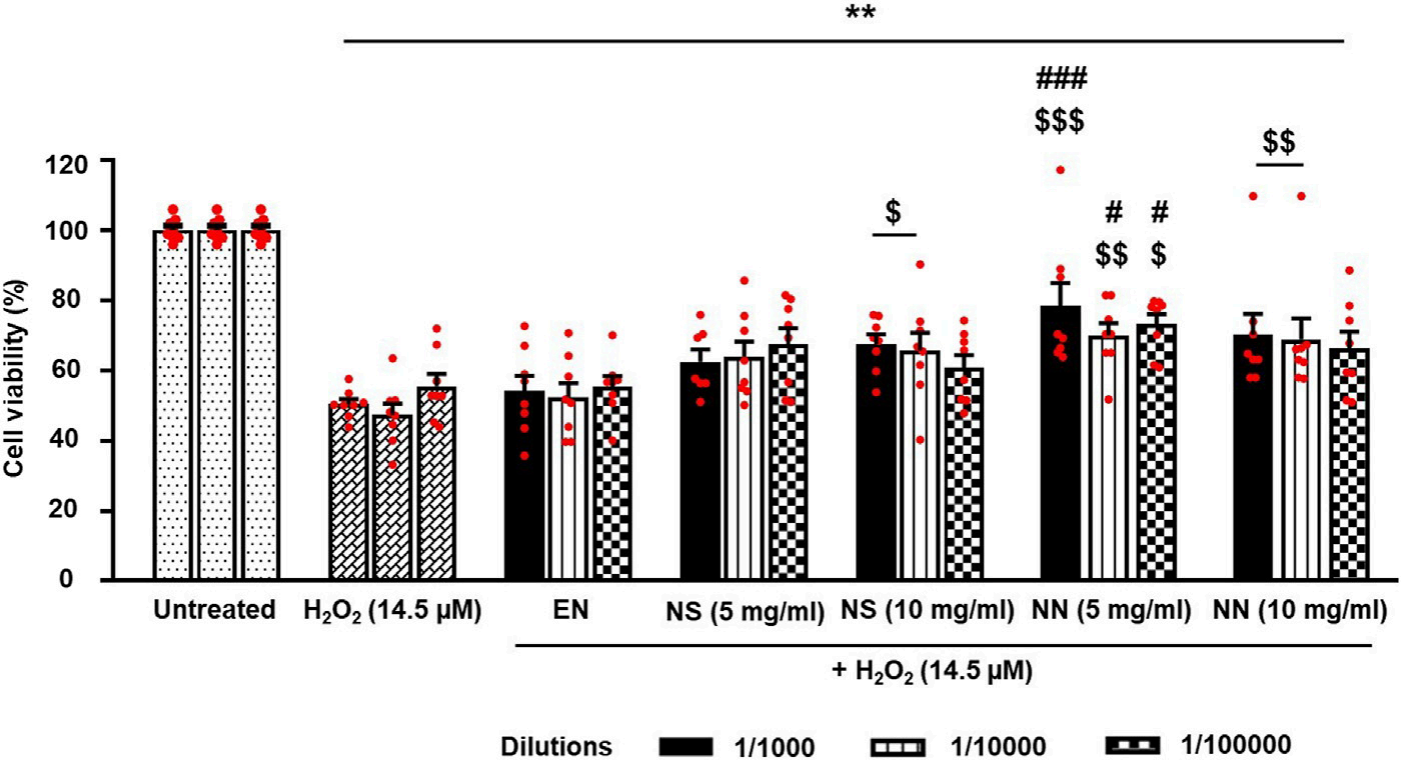
Protective effect of the NN and NS on hydrogen peroxide (H_2_O_2_)-induced oxidative stress in cultured Caco-2 cells. The histogram indicates the viability of Caco-2 cells treated with either H_2_O_2_ (14.5 µM) alone or in combination with either EN, NS (5 or 10 mg/ml), or NN (5 or 10 mg/ml). EN, NS, and NN were tested at three different dilutions (1:1,000, 1:10,000, and 1:100,000). Here, 5 and 10 mg/ml of NN and NS indicate the amount of Nasco pomace loaded inside the nutriosomes and suspension. The data (cell viability, measured by MTT assay) were normalized and expressed as a percentage of the untreated cells (100%). The data are represented as mean ± SEM (*n* = 3). **p < 0.01 vs. untreated; ^$^ *p* < 0.05, ^$$^ *p* < 0.01, ^$$$^ *p* < 0.001 vs. H_2_O_2_ (14.5 µM); #*p* < 0.05, ###*p* < 0.001 vs. EN. Abbreviations: EN, empty nutriosomes; NN, Nasco nutriosomes; NS, Nasco suspension.

Owing to the presence of several antioxidant polyphenols evidenced by the characterization of NPE (Figure 4), we further investigated the protective effect of NS and NN in the counteraction of H_2_O_2_-induced oxidative stress in Caco-2 cells (Kaczara et al., 2010). Firstly, Caco-2 cells were treated with H_2_O_2_ (14.5 µM) either alone or in combination with three different dilutions (1:1,000, 1:10,000, and 1:100,000) of EN, NS (5 or 10 mg/ml), or NN (5 or 10 mg/ml) (Figure 4 **)**.

Two-way ANOVA of Caco-2 cell viability revealed a significant effect of treatment (F_6,146_ = 50.76, *p* < 0.0001), but not of dilution (F_2,146_ = 0.54, *p* = 0.581), or treatment × dilution interaction (F_12,146_ = 0.55, *p* = 0.878). Tukey’s *post hoc* test indicated that exposure of Caco-2 cells to H_2_O_2_ led to a significant reduction in cell viability (∼50%) compared with untreated cells (Figure 4). Treatment with NN (5 mg/ml) significantly contrasted H_2_O_2_-induced reduction in the cell viability at all tested dilutions. Similarly, treatment with NN (10 mg/ml) also protected Caco-2 cells against H_2_O_2_ toxicity, but only when diluted to 1:1,000 or 1:10,000 (Figure 4). Of note, while NS (5 mg/ml) did not exert any cellular protection at any of the dilutions tested, NS (10 mg/ml) significantly protected Caco-2 cells from H_2_O_2_-induced oxidative damage when diluted to 1:1,000 or 1:10,000. To summarize, NPE conferred protection against H_2_O_2_-induced oxidative stress, especially when delivered through the nutriosomes compared with its suspension.

### Immunoreactivity of TH in the CPu and SNc following subacute MPTP treatment and 100 mg/kg of NPE in mice

**FIGURE 5:**
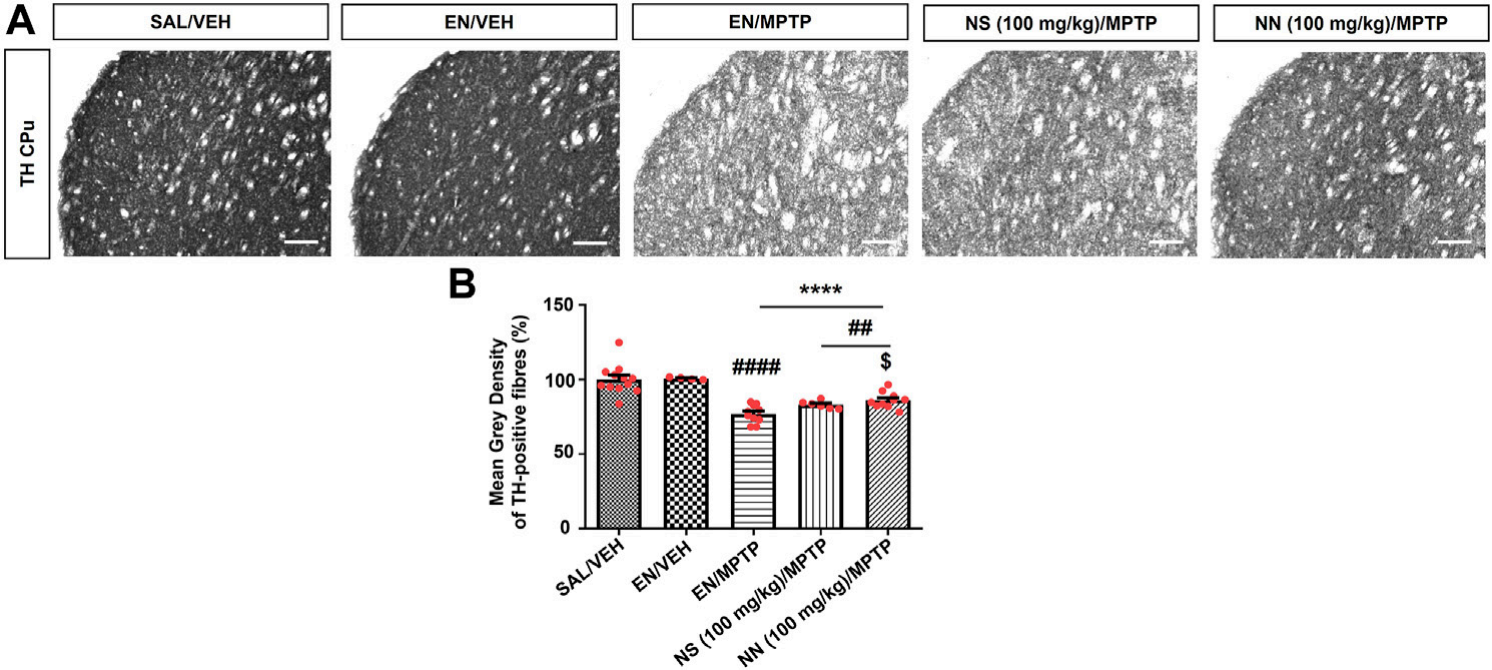
Effect of NN (100 mg/kg) and NS (100 mg/kg) on the immunoreactivity of tyrosine hydroxylase (TH)-positive fibres in the caudate-putamen (CPu) of 1-methyl-4-phenyl-1,2,3,6-tetrahydropyridine (MPTP)-treated mice. **(A)** Representative sections of CPu immunostained for TH. **(B)** Histogram indicates the mean grey density of TH-immunoreactive fibres in the CPu. Values are expressed as a percentage of the SAL/VEH group. Symbols within bars indicate the values of individual mice. ****p < 0.0001 vs. SAL/VEH; ^##^ *p* < 0.01, ^####^ *p* < 0.0001 vs. EN/VEH; ^$^ *p* < 0.05 vs. EN/MPTP. Scale bar = 50 μm. Abbreviations: EN, empty nutriosomes; NN = Nasco nutriosomes; NS, Nasco suspension; SAL, saline; VEH, distilled water.

**FIGURE 6:**
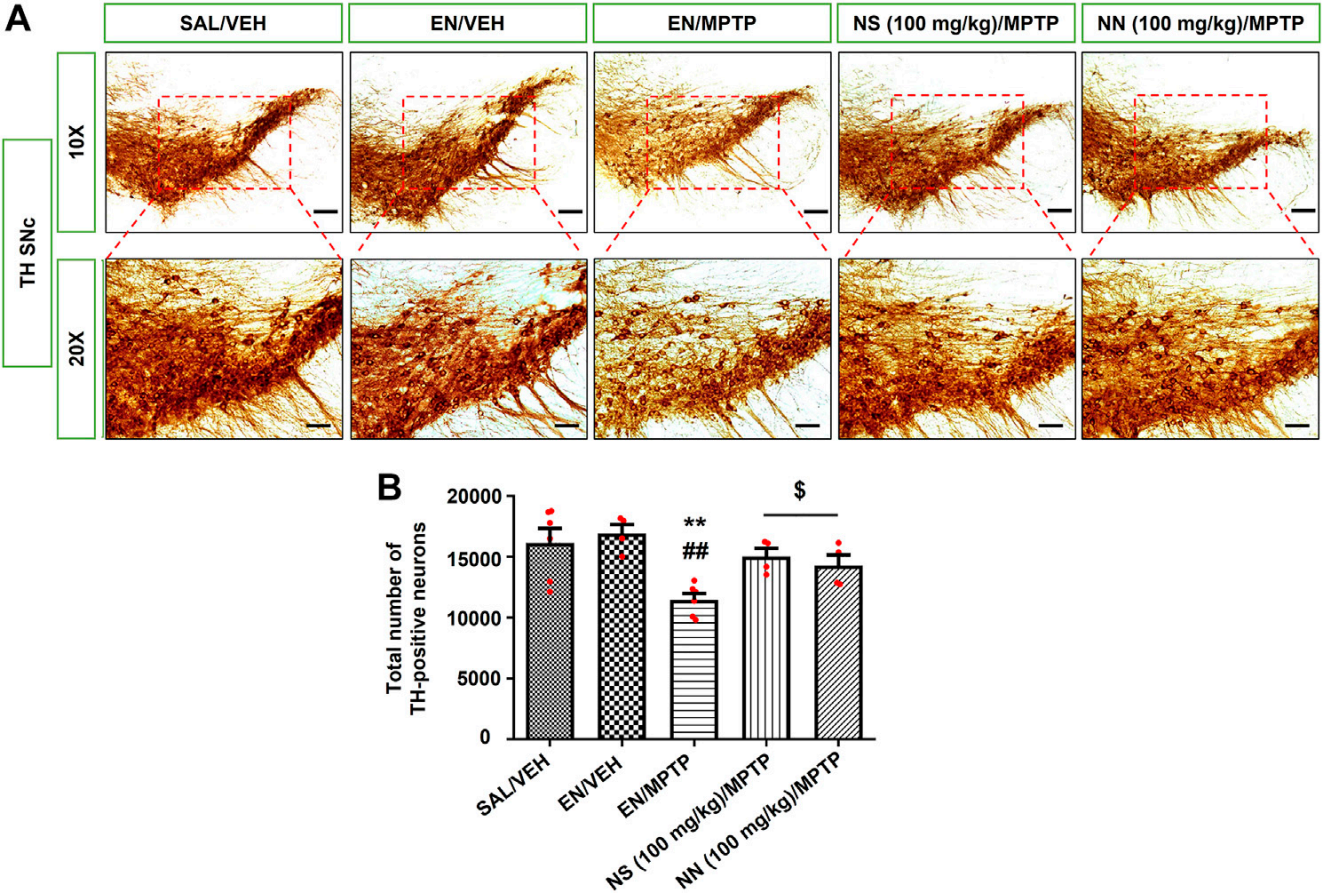
Effect of NN (100 mg/kg) and NS (100 mg/kg) on the immunoreactivity of tyrosine hydroxylase (TH) in the substantia nigra pars compacta (SNc) of 1-methyl-4-phenyl-1,2,3,6-tetrahydropyridine (MPTP)-treated mice. **(A)** Representative sections of SNc immunostained for TH and acquired at 10x (top) and 20x (bottom). **(B)** The histogram indicates the total number of TH-positive cells in the SNc. **p < 0.01 vs. SAL/VEH; ^##^ *p* < 0.01 vs. EN/VEH; ^$^ *p* < 0.05 vs. EN/MPTP. Symbols within bars indicate the values of individual mice. Scale bar = 100 μm (10x) and 50 μm (20x). Abbreviations: EN, empty nutriosomes; NN, Nasco nutriosomes; NS, Nasco suspension; SAL, saline; VEH, distilled water.

To investigate the neuroprotective effect of NN and NS, we employed the subacute MPTP mouse model of PD, wherein mice received the subacute administration of MPTP (20 mg/kg/day × 4, i.p.). Herein, NS served as a reference to evaluate the beneficial effect, as a neuroprotectant, of the drug delivery system of NN. Subacute MPTP treatment significantly reduced the density of TH-positive fibres in the CPu (∼23%, Figure 5) and the number of TH-positive cells in the SNc (∼29%, Figure 6), consistent with earlier findings (Costa et al., 2013).

One-way ANOVA of TH immunoreactivity in experiment 1 (100 mg/kg of NPE) revealed a significant effect of treatment in both the CPu (F_3,32_ = 22.1, *p* ≤ 0.0001) and the SNc (F_4,20_ = 6.343, *p* = 0.0020). In the CPu, Newman–Keuls *post hoc* test indicated a significant reduction in the mean grey intensity of TH-positive fibres in mice receiving either EN/MPTP, NS (100 mg/kg)/MPTP, or NN (100 mg/kg)/MPTP compared with both SAL/VEH- and EN/VEH-treated mice (Figure 5B). Of note, NS (100 mg/kg)/MPTP treatment did not modify the mean grey intensity of TH-positive fibres compared with EN/MPTP (Figure 5B), while NN (100 mg/kg)/MPTP treatment significantly counteracted the reduction in the mean grey intensity of dopaminergic TH-positive fibres observed in EN/MPTP-treated mice (Figure 5B).

In the SNc, Newman–Keuls post-hoc test indicated a significant reduction in the total number of TH-positive neurons in mice receiving EN/MPTP compared with both SAL/VEH- and EN/VEH-treated mice (Figure 6B). Importantly, both NN (100 mg/kg)/MPTP and NS (100 mg/kg)/MPTP treatment significantly counteracted the reduction in the total number of TH-positive neurons observed in EN/MPTP-treated mice (Figure 6B).

### Immunoreactivity of DAT in the CPu following subacute MPTP treatment and 100 mg/kg of NPE in mice

**FIGURE 7:**
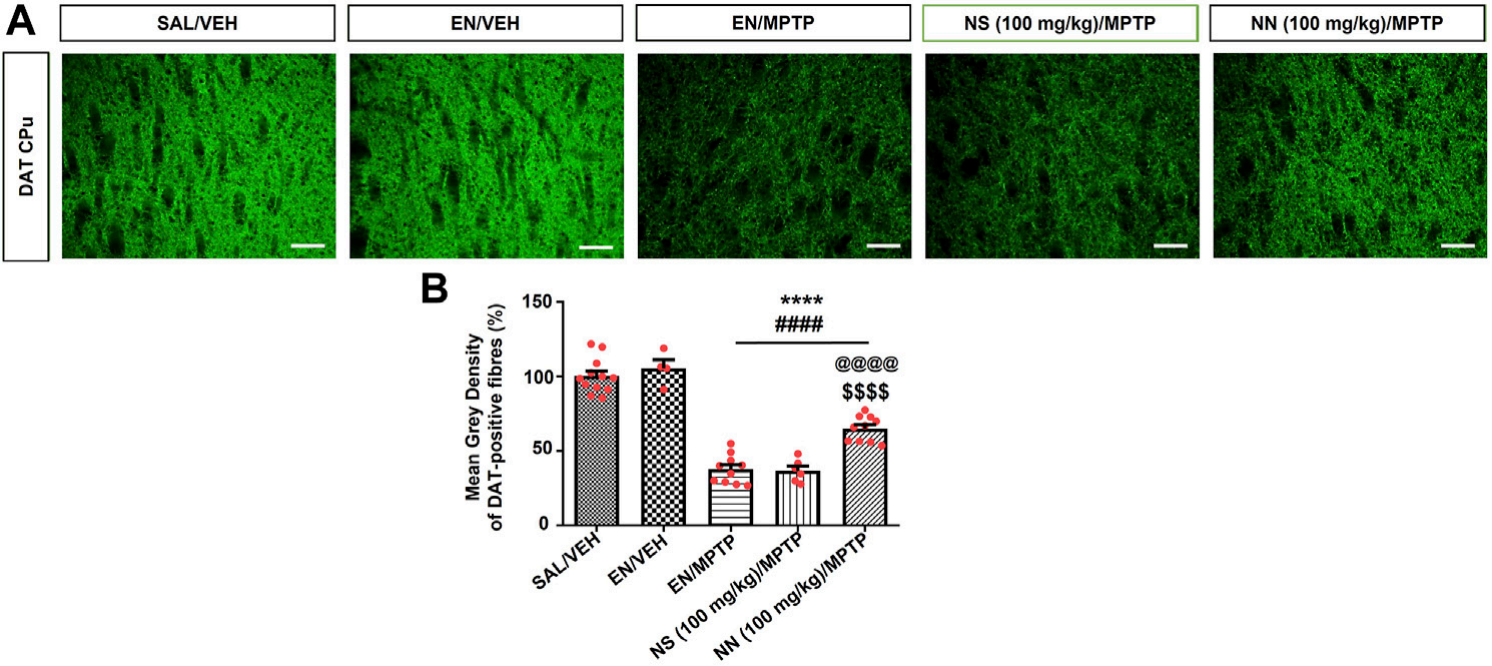
Effect of NN (100 mg/kg) and NS (100 mg/kg) on the immunoreactivity of dopamine transporter (DAT) in the caudate-putamen (CPu) of 1-methyl-4-phenyl-1,2,3,6-tetrahydropyridine (MPTP)-treated mice. **(A)** Representative sections of CPu immunostained for DAT. **(B)** The histograms indicate the mean grey density of DAT-immunoreactive fibres in the CPu. Values are expressed as a percentage of the SAL/VEH group. Symbols within bars indicate the values of individual mice. ****p < 0.0001 vs. SAL/VEH; ^####^ *p* < 0.0001 vs. EN/VEH; ^$$$$^ *p* < 0.0001 vs. EN/MPTP; ^@@@@^ *p* < 0.0001 vs. NS (100 mg/kg)/MPTP. Scale bar = 50 μm. Abbreviations: EN, empty nutriosomes; NN, Nasco nutriosomes; NS, Nasco suspension; SAL, saline; VEH, distilled water.

DAT is a plasma membrane protein located in dopaminergic terminals, where it regulates synaptic dopamine (DA) levels and therefore provides a useful marker of functional activity of dopaminergic neurons. Indeed, many neuroimaging studies evidenced the reduced density of DAT in the brain of PD patients. Therefore, we have also evaluated the expression of striatal DAT and found that subacute MPTP treatment significantly reduced the density of DAT-positive fibres in the CPu (∼62%, Figure 7).

One-way ANOVA of DAT immunoreactivity in experiment 1 (100 mg/kg of NPE) revealed a significant effect of treatment in the CPu (F_4,38_ = 81.9, *p* < 0.0001). Newman–Keuls *post hoc* test indicated a significant reduction in the mean grey intensity of DAT-positive fibres in mice receiving either EN/MPTP, NS (100 mg/kg)/MPTP, or NN (100 mg/kg)/MPTP compared with both SAL/VEH- and EN/VEH-treated mice (Figure 7B). Of note, NS (100 mg/kg)/MPTP treatment did not modify the intensity of DAT-positive fibres compared with EN/MPTP (Figure 7B), while NN (100 mg/kg)/MPTP treatment significantly counteracted the loss of DAT-positive fibres compared with EN/MPTP (Figure 7B).

### Immunoreactivity of TH in the CPu and SNc, and of DAT in the CPu following subacute MPTP treatment and 50 mg/kg of NPE in mice

Considering the promising results obtained with NN (100 mg/kg), we next asked whether NPE might still be neuroprotective toward MPTP dopaminergic neurotoxicity at the lower dose of 50 mg/kg.

Results of TH immunoreactivity in experiment 2 (50 mg/kg of NPE) revealed a significant effect of treatment in the CPu (one-way ANOVA, F_3,12_ = 39.9, *p* < 0.0001), and the SNc (F_3,12_ = 31.02, *p* < 0.0001) (Table 1). In the CPu, Newman–Keuls *post hoc* test indicated that the mean grey intensity of TH-positive fibres was statistically decreased in mice receiving either EN/MPTP or NN (50 mg/kg)/MPTP treatment compared with SAL/VEH (Table 1). Importantly, NN (50 mg/kg)/MPTP treatment was unable to counteract the reduction in TH-positive fibres observed in EN/MPTP-treated mice (Table 1).

**TABLE 1:** Effect of NN (50 mg/kg) on the immunoreactivity of tyrosine hydroxylase (TH)-positive fibres in the caudate-putamen (CPu), TH-positive cells in the substantia nigra pars compacta (SNc), and dopamine transporter (DAT)-positive fibres in the CPu of 1-methyl-4-phenyl-1,2,3,6-tetrahydropyridine (MPTP)-treated mice.

Groups	% of TH-positive fibres (CPu)	Number of TH-positive cells (SNc)	% of DAT-positive fibres (CPu)
SAL/VEH	100 ± 1.2	17,860 ± 637	100 ± 5.9
EN/VEH	101 ± 0.4	18,878 ± 469	105.5 ± 5.6
EN/MPTP	81.7 ± 2.4^****^ ^,^ ^####^	11,521 ± 652^**^ ^,^ ^##^	42.1 ± 2.1^****^ ^,^ ^####^
NN (50 mg/kg)/MPTP	83.4 ± 1.8^****^ ^,^ ^####^	13,445 ± 735^**^ ^,^ ^##^	38.5 ± 4.9^****^ ^,^ ^####^

** *p* < 0.01.

**** *p* < 0.0001 vs. SAL/VEH.

## *p* < 0.01.

#### *p* < 0.0001 vs. EN/VEH. *n* = 4/group. Abbreviations: EN, empty nutriosomes; NN, nasco nutriosomes; SAL, saline; VEH, distilled water.

Similarly, in the SNc, Newman–Keuls *post hoc* test indicated that the total number of TH-positive neurons was statistically decreased in mice receiving either EN/MPTP or NN (50 mg/kg)/MPTP treatment compared with SAL/VEH- and EN/VEH-treated mice (Table 1). Of note, NN (50 mg/kg)/MPTP treatment was unable to counteract the reduction in TH-positive neurons observed in EN/MPTP-treated mice (Table 1).

Results of DAT immunoreactivity in experiment 2 (50 mg/kg of NPE) revealed a significant effect of treatment in the CPu (F_3,12_ = 54, *p*<0.0001). Newman–Keuls *post hoc* test indicated that the mean grey intensity of DAT-positive fibres was statistically reduced in the mice receiving EN/MPTP or NN (50 mg/kg)/MPTP treatment compared with SAL/VEH (Table 1). In line with TH immunoreactivity analysis in the CPu, NN (50 mg/kg)/MPTP treatment was unable to counteract the demise of DAT-positive fibres observed in EN/MPTP-treated mice (Table 1 **)**.

## Discussion

The most important finding of the present study was that NN and, to a significantly lesser extent, NS, did prevent the degeneration of nigro-striatal dopaminergic neurons produced in mice by subacute administration of MPTP, a neurotoxin known to induce PD in humans. Considering that, at the moment, the therapy of PD is exclusively symptomatic, the present findings add an important perspective to the preventive neuroprotective therapy of PD.

Moreover, the performed characterization of NN components demonstrated the richness of Nasco pomace in bioactive polyphenols showing, in addition, its high stability, biocompatibility, and antioxidant potential.

## Richness of NPE in antioxidant polyphenols

According to the World Health Organizition, the medical use of plant-based natural products is growing remarkably (Khan and Ahmad, 2019). By providing a wide range of bioactive compounds, plant-based products constitute a useful resource for the development of new therapeutics for the management of multiple pathologies, including PD (Shahpiri et al., 2016). In this respect, despite the huge need of clinical validation, several plant-based extracts (e.g., *Bacopa monnieri, Mucuna pruriens, Withania somnifera*) and phytochemicals (e.g., baicalein, curcumin, resveratrol, epigallocatechin gallate) demonstrated to have neuroprotective effects both *in vitro* and *in vivo* models of PD (Kasture et al., 2009; Srivastav et al., 2017; Javed et al., 2019).

In this context, grapes have recently received much interest because they are rich natural sources of antioxidant polyphenols (i.e., phenolic acids, flavan-3-ols, flavonols, etc.) with potential therapeutic applications (Suleria et al., 2020). Interestingly, although grape pomaces are considered a waste by-product, they still possess high amounts of several polyphenols that possess higher therapeutic value (Manca et al., 2020).

The qualitative analysis of Nasco pomace demonstrated the presence of five phenolic compounds: gallic acid, (+)-catechin, (−)-epicatechin, procyanidin B2, and quercetin. Of note, the quantitative analysis indicated the highest abundance of procyanidin B2 followed by (+)-catechin, quercetin, and (−)-epicatechin, while gallic acid was present in the lowest amount. To this end, *in vivo* studies carried out using the unilateral 6-OHDA model of PD have advocated the anti-inflammatory, antioxidative, and neuroprotective potential of the above-mentioned individual polyphenols. In that context, systemic administration of catechin to 6-OHDA-lesioned rats not only rescued motor and memory deficits but also significantly contrasted the 6-OHDA-induced decrease in mesencephalic DA levels and TH immunoreactivity in the nigro-striatal dopaminergic neurons (Teixeira et al., 2013). In addition, Zhang et al. (2019) demonstrated the protective role of procyanidin B2 in 6-OHDA lesioned rats at both the *in vivo* (motor behavior) and *in vitro* (cell viability, mitochondrial membrane potential, and total superoxide dismutase changes) levels. Therefore, the two phenolic compounds most abundant in Nasco pomace have displayed an interesting protective role in the acute 6-OHDA model of PD.

## Neuroprotective effect of NN and NS in the subacute MPTP mouse model

Although the findings reported above evidenced the neuroprotective effects exerted by individual polyphenols, the above-mentioned studies employed the unilateral 6-OHDA mouse model of PD, a preclinical model in which the degeneration of the nigro-striatal dopaminergic neurons is rapid, massive, and located only in one side of the brain, which is very different from the type of degeneration that has been observed in idiopathic PD. In our study, we utilized a neurodegenerative model of PD that employed the subacute MPTP mouse model of PD, a model known to produce a more progressive and bilateral degeneration of nigro-striatal dopaminergic neurons. Moreover, MPTP has been reported to cause PD in humans, and several studies have shown the ability of the MPTP mouse model to replicate the pathophysiological events that characterize PD (Meredith and Rademacher, 2011). We can therefore conclude that the results reported here may show a predictive neuroprotective efficacy of the Nasco pomace when incorporated into nutriosomes in a model that more closely reproduces the human PD pathology.

Neuroprotection against MPTP-induced neurotoxicity in mice was evaluated by immunoreactivity of TH, the rate-limiting enzyme of DA biosynthesis, in the CPu and SNc and, DAT in the CPu. In agreement with previous findings from our group (Frau et al., 2011; Costa et al., 2013), mice receiving a subacute regimen of MPTP (20 mg/kg/day, for 4 days, i.p.) showed a significant reduction in the density of TH-positive fibres in the CPu and the number of TH-positive cells in the SNc, indicating a marked loss of nigro-striatal dopaminergic neurons.

Of great interest, repeated combined treatment of MPTP with NN (100 mg/kg) conferred significant neuroprotection on the TH immunoreactivity, whereas treatment with a lower dose of NN (50 mg/kg) failed to counteract MPTP-induced loss of TH immunoreactivity in both the CPu and SNc, showing a dose-response efficacy.

Interestingly, the results of the present study also showed limited efficacy of NS compared with NN, since the neuroprotective effects on TH immunoreactivity exerted by NS (100 mg/kg) were limited to the SNc. In this respect, it is important to consider previous findings indicating that dopaminergic fibres in the CPu, and dopaminergic somas in the SNc, possess a different sensitivity towards MPTP neurotoxicity (Huang et al., 2017), which is not directly correlated to the local concentration of 1-methyl-4-phenyl-2,3-dihydropyridinium ion (MPP^+^), the toxic metabolite of MPTP (Vaglini et al., 1996). Thus, while in the SNc, NN (100 mg/kg) and NS (100 mg/kg) equally counteracted the MPTP-induced loss of TH immunoreactivity, in the CPu, we speculate that the higher bioavailability ensured by nutriosomes over the suspension was essential to contrast MPTP neurotoxicity more effectively at the level of striatal dopaminergic terminals. Furthermore, our results suggest that striatal levels of DAT, the main index of the functionality of dopaminergic terminals, are more sensitive than TH to the MPTP effect and to the neuroprotective efficacy of NN. In fact, evaluation of the immunoreactivity for the DAT in the CPu showed a net reduction in the density of DAT-positive fibres in the CPu of mice receiving a subacute MPTP-treatment, which was effectively counteracted by NN, but not NS, at the dose of 100 mg/kg. Importantly, this result was dose-dependent as NN (50 mg/kg) did not significantly counteract the decrease in DAT immunoreactivity.

Overall, the immunohistochemistry results support a superior neuroprotective potential of NN over NS against MPTP-induced neurotoxicity of the dopaminergic system, thus highlighting the importance of oral administration of NPE in nutriosomes over suspension.

Degeneration of nigro-striatal dopaminergic neurons is a pathological hallmark of PD, which leads to DA deficiency in the CPu and the development of the cardinal motor symptoms of PD (Poewe et al., 2017). Therefore, the neuroprotective efficacy of NN on DA neuron degeneration in a subacute MPTP mouse model of PD suggests an important utilization of NN to prevent and delay the onset of this disease.

## Bioavailability, biocompatibility, and antioxidant potential of NN

A very important finding of the present study is the demonstration of the higher efficacy of NN over NS. Although grape pomaces are rich in polyphenols, major problems are associated with their therapeutic use due to their low water solubility, extensive degradation in the gastrointestinal tract, reduced blood-brain barrier (BBB) penetration, and consequently low bioavailability at the target sites, when administered orally. Therefore, our results emphasize how important it is to incorporate polyphenol-rich compounds into nanovesicles to overcome the above-mentioned problems associated with their oral use (Manconi et al., 2020).

In this context, nano-incorporation of curcumin (a flavonoid-rich antioxidant) and piperine (an alkaloid) was found to improve their bioavailability and BBB crossing ability (Kundu et al., 2016). Most importantly, curcumin and piperine-loaded nanovesicles protected nigral dopaminergic neurons against rotenone-induced neurotoxicity by inhibiting the alpha-synuclein aggregation and oxidative stress, thus alleviating the behavioral deficits in the rotenone mouse model of PD (Kundu et al., 2016). Similarly, resveratrol (a stilbenoid polyphenol)-loaded liposomes protected dopaminergic neurons against 6-OHDA-induced oxidative stress and alleviated behavioral impairments in the unilateral 6-OHDA rat model of PD (Wang et al., 2011). Of note, the therapeutic effects produced by resveratrol-loaded liposomes were found to be superior as compared to that of non-loaded resveratrol, likely due to its increased bioavailability (Wang et al., 2011). Altogether, these evidences highlight the therapeutic advantages given by nanoformulations for the delivery of bioactive polyphenols to the central nervous system.

Taking into consideration the above-mentioned studies, in the present study, we developed innovative phospholipid-based nanovesicles known as nutriosomes, containing the highest amount of Nutriose^®^ (a soluble, prebiotic fiber) (Catalán-Latorre et al., 2018), which, in addition, has been shown to improve human gut-microbiota composition and reduce blood glucose levels, if consumed daily (Lefranc-Millot, 2008). Being formulations tailored for intragastric administration and intended to pass the intestinal wall, the biocompatibility displayed by NN using Caco-2 cells, which showed a significant increase in cell viability, is of great relevance. These cells, in fact, differentiate into a monolayer of polarized cells, coupled by tight junctions, which express many morpho-functional characteristics of the absorbing epithelium of the small intestine (Angelis and Turco, 2011). In this regard, previously curcumin showed higher plasma bioavailability when incorporated inside nutriosomes compared with its suspension (Catalán-Latorre et al., 2018). The nutriosome-mediated improvement of curcumin pharmacokinetics resulted in a better counteraction of colitis, in the 2,4,6-trinitrobenzene sulfonic acid rat model, compared with curcumin suspension (Catalán-Latorre et al., 2018). In addition, the suspension of grape pomace obtained from *Vitis vinifera* (variety cannonau) produced inferior antioxidative effects in Caco-2 cells exposed to H_2_O_2_ compared with cannonau nutriosomes (Allaw et al., 2020). In this context, our study also demonstrated that both NN and NS significantly contrasted H_2_O_2_-induced oxidative damage in Caco-2 cells. However, the protective effect exerted by NS was inferior compared with NN, which further highlights the importance of nutriosomes over suspension. Overall, these lines of evidence further confirm the ability of nanoformulations to improve the bioavailability, biodistribution, and therapeutic effects of specific compounds when administered orally. Therefore, our results suggest that nutriosomes protect the antioxidant polyphenols present in the Nasco pomace and increase their bioavailability at the target sites, rendering them more effective in protecting against MPTP-induced damage to the nigro-striatal DA neurons.

A recent study investigated the protective effect of liposome-loaded polyphenol-rich grape pomace extract in an *in vitro* rotenone-based model of PD (Marino et al., 2021). Of note, consistent with our data, the findings demonstrated that grape pomace-loaded-liposomes completely rescued rotenone-induced oxidative stress and alpha-synuclein aggregation in SH-SH5Y cells. However, while this study was performed in *vitro* preparations, our results added an important finding to the utility of grape pomace-loaded nutriosomes, since the neuroprotective effects were obtained using an *in vivo* subacute animal model of MPTP.

In conclusion, nutriosomes loaded with NPE rich in antioxidant polyphenols significantly protected nigro-striatal dopaminergic neurons in a subacute MPTP mouse model of PD, suggesting a therapeutic application for grape pomaces generated as a result of wine production. In addition, considering that wine production is linked to the generation of a large volume of pomaces, this finding may also help to reduce the waste burden of grape pomaces on the environment. Future studies are now being focused on investigating the effects of NN in MPTP-induced neuroinflammation and oxidative stress in mice, as these are the most important events known to trigger neurodegeneration (de Araújo et al., 2021).

## Data Availability

The original contributions presented in the study are included in the article/Supplementary Material, further inquiries can be directed to the corresponding author.

## References

[B1] Allaw M. Manca M. L. Caddeo C. Recio M. C. Pérez-Brocal V. Moya A. (2020). Advanced strategy to exploit wine-making waste by manufacturing antioxidant and prebiotic fibre-enriched vesicles for intestinal health. Colloids Surf. B Biointerfaces 193, 111146. 10.1016/j.colsurfb.2020.111146 32485579

[B2] Angelis I. D. Turco L. (2011). Caco2 cells as a model for intestinal absorption. Curr. Protoc. Toxicol. 47, Unit20.6. 10.1002/0471140856.tx2006s47 21400683

[B3] Ascherio A. Schwarzschild M. A. (2016). The epidemiology of Parkinson's disease: risk factors and prevention. Lancet. Neurol. 15, 1257–1272. 10.1016/s1474-4422(16)30230-7 27751556

[B4] Azwanida N. (2015). A review on the extraction methods use in medicinal plants, principle, strength and limitation. Med. Aromat. Plants 4, 2167–0412. 10.4172/2167-0412.1000196

[B5] Ben Youssef S. Brisson G. Doucet-Beaupré H. Castonguay A.-M. Gora C. Amri M. (2021). Neuroprotective benefits of grape seed and skin extract in a mouse model of Parkinson’s disease. Nutr. Neurosci. 24, 197–211. 10.1080/1028415X.2019.1616435 31131731

[B6] Bitu Pinto N. da Silva Alexandre B. Neves K. R. T. Silva A. H. Leal L. K. A. Viana G. S. (2015). Neuroprotective properties of the standardized extract from Camellia sinensis (green tea) and its main bioactive components, epicatechin and epigallocatechin gallate, in the 6-OHDA model of Parkinson’s disease. Evid. Based. Complement. Altern. Med. 2015, 161092. 10.1155/2015/161092 PMC4488543 26167188

[B7] Bové J. Perier C. (2012). Neurotoxin-based models of Parkinson's disease. Neuroscience 211, 51–76. 10.1016/j.neuroscience.2011.10.057 22108613

[B8] Caldas T. W. Mazza K. E. Teles A. S. Mattos G. N. Brígida A. I. S. Conte-Junior C. A. (2018). Phenolic compounds recovery from grape skin using conventional and non-conventional extraction methods. Ind. Crops Prod. 111, 86–91. 10.1016/j.indcrop.2017.10.012

[B9] Castangia I. Nácher A. Caddeo C. Merino V. Díez-Sales O. Catalán-Latorre A. (2015). Therapeutic efficacy of quercetin enzyme-responsive nanovesicles for the treatment of experimental colitis in rats. Acta Biomater. 13, 216–227. 10.1016/j.actbio.2014.11.017 25463498

[B10] Casu M. A. Pisu C. Lobina C. Pani L. (2004). Immunocytochemical study of the forebrain serotonergic innervation in Sardinian alcohol-preferring rats. Psychopharmacology 172, 341–351. 10.1007/s00213-003-1663-z 14634717

[B11] Catalán-Latorre A. Pleguezuelos-Villa M. Castangia I. Manca M. L. Caddeo C. Nácher A. (2018). Nutriosomes: prebiotic delivery systems combining phospholipids, a soluble dextrin and curcumin to counteract intestinal oxidative stress and inflammation. Nanoscale 10, 1957–1969. 10.1039/C7NR05929A 29319093

[B12] Costa G. Frau L. Wardas J. Pinna A. Plumitallo A. Morelli M. (2013). MPTP‐induced dopamine neuron degeneration and glia activation is potentiated in MDMA‐pretreated mice. Mov. Disord. 28, 1957–1965. 10.1002/mds.25646 24108425

[B13] Costa G. Morelli M. Simola N. (2017). Progression and persistence of neurotoxicity induced by MDMA in dopaminergic regions of the mouse brain and association with noradrenergic, GABAergic, and serotonergic damage. Neurotox. Res. 32, 563–574. 10.1007/s12640-017-9761-6 28597409

[B14] Costa G. Porceddu P. F. Serra M. Casu M. A. Schiano V. Napolitano F. (2019). Lack of Rhes increases MDMA-induced neuroinflammation and dopamine neuron degeneration: Role of gender and age. Int. J. Mol. Sci. 20, 1556. 10.3390/IJMS20071556 PMC6480667 30925704

[B15] Costa G. Simola N. Morelli M. (2014). MDMA administration during adolescence exacerbates MPTP-induced cognitive impairment and neuroinflammation in the hippocampus and prefrontal cortex. Psychopharmacology 231, 4007–4018. 10.1007/S00213-014-3536-Z 24687411

[B16] de Araújo F. M. Cuenca-Bermejo L. Fernández-Villalba E. Costa S. L. Silva V. D. A. Herrero M. T. (2021). Role of microgliosis and NLRP3 inflammasome in Parkinson’s disease pathogenesis and therapy. Cell. Mol. Neurobiol. 1, 1283–1300. 10.1007/S10571-020-01027-6 PMC11421755 33387119

[B18] Fenu S. Wardas J. Morelli M. (2009). Impulse control disorders and dopamine dysregulation syndrome associated with dopamine agonist therapy in Parkinson's disease. Behav. Pharmacol. 20, 363–379. 10.1097/FBP.0B013E32833109A0 19724195

[B19] Frau L. Borsini F. Wardas J. Khairnar A. S. Schintu N. Morelli M. (2011). Neuroprotective and anti‐inflammatory effects of the adenosine A2A receptor antagonist ST1535 in a MPTP mouse model of Parkinson's disease. Synapse 65, 181–188. 10.1002/SYN.20833 20665698

[B20] Grgić J. Šelo G. Planinić M. Tišma M. Bucić-Kojić A. (2020). Role of the encapsulation in bioavailability of phenolic compounds. Antioxidants 9, 923. 10.3390/ANTIOX9100923 PMC7601682 32993196

[B21] Gupta M. Dey S. Marbaniang D. Pal P. Ray S. Mazumder B. (2020). Grape seed extract: Having a potential health benefits. J. Food Sci. Technol. 57 (4), 1205–1215. 10.1007/s13197-019-04113-w 32180617 PMC7054588

[B22] Huang D. Xu J. Wang J. Tong J. Bai X. Li H. (2017). Dynamic changes in the nigrostriatal pathway in the MPTP mouse model of Parkinson’s disease. Parkinson's Dis. 2017, 9349487. 10.1155/2017/9349487 28831326 PMC5555011

[B23] Javed H. Nagoor Meeran M. F. Azimullah S. Adem A. Sadek B. Ojha S. K. (2019). Plant extracts and phytochemicals targeting α-synuclein aggregation in Parkinson's disease models. Front. Pharmacol. 9, 1555. 10.3389/fphar.2018.01555 30941047 PMC6433754

[B24] Kaczara P. Sarna T. Burke J. M. (2010). Dynamics of H2O2 availability to ARPE-19 cultures in models of oxidative stress. Free Radic. Biol. Med. 48, 1064–1070. 10.1016/J.FREERADBIOMED.2010.01.022 20100568 PMC2839027

[B25] Kasture S. Pontis S. Pinna A. Schintu N. Spina L. Longoni R. (2009). Assessment of symptomatic and neuroprotective efficacy of Mucuna pruriens seed extract in rodent model of Parkinson’s disease. Neurotox. Res. 15 (2), 111–122. 10.1007/s12640-009-9011-7 19384573

[B26] Kavitha R. V. Kumar J. Egbuna C. Ifemeje J. C. (2020). Phytochemicals as therapeutic interventions in neurodegenerative diseases. Phytochemicals as Lead Compd. New Drug Discov. 2020, 161–178. 10.1016/B978-0-12-817890-4.00010-X

[B27] Khan M. S. A. Ahmad I. (2019). Herbal medicine: current trends and future prospects. New look phytomedicine 2019, 3–13. 10.1016/B978-0-12-814619-4.00001-X

[B28] Kundu P. Das M. Tripathy K. Sahoo S. K. (2016). Delivery of dual drug loaded lipid based nanoparticles across the blood–brain barrier impart enhanced neuroprotection in a rotenone induced mouse model of Parkinson’s disease. ACS Chem. Neurosci. 7 (12), 1658–1670. 10.1021/acschemneuro.6b00207 27642670

[B29] Lacombe T. Audeguin L. Boselli M. Bucchetti B. Cabello F. Chatelet P. (2011). Grapevine European catalogue: Towards a comprehensive list. Vitis -Geilweilerhof 50 (2), 65–68.

[B30] Langston J. W. Ballard P. Tetrud J. W. Irwin I. (1983). Chronic Parkinsonism in humans due to a product of meperidine-analog synthesis. Science 219, 979–980. 10.1126/SCIENCE.6823561 6823561

[B31] Lefranc-Millot C. (2008). NUTRIOSE® 06: a useful soluble dietary fibre for added nutritional value. Nutr. Bull. 33, 234–239. 10.1111/J.1467-3010.2008.00711.X

[B32] Maier T. Fromm M. Schieber A. Kammerer D. R. Carle R. (2009). Process and storage stability of anthocyanins and non-anthocyanin phenolics in pectin and gelatin gels enriched with grape pomace extracts. Eur. Food Res. Technol. 229, 949–960. 10.1007/S00217-009-1134-9

[B33] Manca M. L. Casula E. Marongiu F. Bacchetta G. Sarais G. Zaru M. (2020). From waste to health: Sustainable exploitation of grape pomace seed extract to manufacture antioxidant, regenerative and prebiotic nanovesicles within circular economy. Sci. Rep. 10, 14184. 10.1038/s41598-020-71191-8 32843707 PMC7447760

[B34] Manconi M. Caddeo C. Manca M. L. Fadda A. M. (2020). Oral delivery of natural compounds by phospholipid vesicles. Nanomedicine 15, 1795–1803. 10.2217/NNM-2020-0085 32698672

[B35] Marino A. Battaglini M. Desii A. Lavarello C. Genchi G. Petretto A. (2021). Liposomes loaded with polyphenol-rich grape pomace extracts protect from neurodegeneration in a rotenone-based *in vitro* model of Parkinson's disease. Biomater. Sci. 9, 8171–8188. 10.1039/D1BM01202A 34617936

[B36] Martí Coma-Cros E. Biosca A. Lantero E. Manca M. L. Caddeo C. Gutiérrez L. (2018). Antimalarial activity of orally administered curcumin incorporated in Eudragit®-containing liposomes. Int. J. Mol. Sci. 19, 1361. 10.3390/IJMS19051361 PMC5983818 29734652

[B37] McDowell K. Chesselet M.-F. (2012). Animal models of the non-motor features of Parkinson's disease. Neurobiol. Dis. 46, 597–606. 10.1016/J.NBD.2011.12.040 22236386 PMC3442929

[B38] Meredith G. E. Rademacher D. J. (2011). MPTP mouse models of Parkinson's disease: an update. J. Park. Dis. 1, 19–33. 10.3233/JPD-2011-11023 PMC3530193 23275799

[B39] Obeso J. Stamelou M. Goetz C. Poewe W. Lang A. Weintraub D. (2017). Past, present, and future of Parkinson's disease: A special essay on the 200th anniversary of the shaking palsy. Mov. Disord. 32, 1264–1310. 10.1002/MDS.27115 28887905 PMC5685546

[B40] Orru M. Grillo O. Venora G. Bacchetta G. (2015). Seed morpho‐colorimetric analysis by computer vision: a helpful tool to identify grapevine (V itis vinifera L.) cultivars. Aust. J. Grape Wine Res. 21 (3), 508–519. 10.1111/ajgw.12153

[B41] Paunović D. Đ. Mitić S. S. Kostić D. A. Mitić M. N. Stojanović B. T. Pavlović J. L. (2014). Kinetics and thermodynamics of the solid-liquid extraction process of total polyphenols from barley. Savr. Tehnol. 3, 58–63. 10.5937/SAVTEH1402058P

[B42] Paxinos G. Franklin K. B. (2008). The mouse brain in stereotaxic coordinates. 3rd edn. Cambridge, Massachusetts, United States: Academic Press.

[B43] Pazos-Tomas C. C. Cruz-Venegas A. Pérez-Santiago A. D. Sánchez-Medina M. A. Matías-Pérez D. García-Montalvo I. A. (2020). Vitis vinifera: An alternative for the prevention of neurodegenerative diseases. J. Oleo Sci. 69, 1147–1161. 10.5650/JOS.ESS20109 32908097

[B44] Poewe W. Seppi K. Tanner C. M. Halliday G. M. Brundin P. Volkmann J. (2017). Parkinson disease. Nat. Rev. Dis. Prim. 3, 17013. 10.1038/NRDP.2017.13 28332488

[B45] Romdhane M. Gourdon C. (2002). Investigation in solid–liquid extraction: influence of ultrasound. Chem. Eng. J. 87, 11–19. 10.1016/S1385-8947(01)00206-6

[B46] Shahpiri Z. Bahramsoltani R. Farzaei M. H. Farzaei F. Rahimi R. (2016). Phytochemicals as future drugs for Parkinson’s disease: a comprehensive review. Rev. Neurosci. 27 (6), 651–668. 10.1515/revneuro-2016-0004 27124673

[B47] Sriraksa N. Wattanathorn J. Muchimapura S. Tiamkao S. Brown K. Chaisiwamongkol K. (2012). Cognitive-enhancing effect of quercetin in a rat model of Parkinson's disease induced by 6-hydroxydopamine. Evid. Based. Complement. Altern. Med. 2012, 823206. 10.1155/2012/823206 PMC3139913 21792372

[B48] Srivastav S. Fatima M. Mondal A. C. (2017). Important medicinal herbs in Parkinson’s disease pharmacotherapy. Biomed. Pharmacother. 92, 856–863. 10.1016/j.biopha.2017.05.137 28599249

[B49] Suleria H. A. Barrow C. J. Dunshea F. R. (2020). Screening and characterization of phenolic compounds and their antioxidant capacity in different fruit peels. Foods 9, 1206. 10.3390/FOODS9091206 PMC7556026 32882848

[B50] Teixeira M. Souza C. Menezes A. Carmo M. Fonteles A. Gurgel J. (2013). Catechin attenuates behavioral neurotoxicity induced by 6-OHDA in rats. Pharmacol. Biochem. Behav. 110, 1–7. 10.1016/J.PBB.2013.05.012 23714698

[B51] Terral J.-F. Tabard E. Bouby L. Ivorra S. Pastor T. Figueiral I. (2010). Evolution and history of grapevine (Vitis vinifera) under domestication: new morphometric perspectives to understand seed domestication syndrome and reveal origins of ancient European cultivars. Ann. Bot. 105 (3), 443–455. 10.1093/aob/mcp298 20034966 PMC2826248

[B52] Vaglini F. Fascetti F. Tedeschi D. Cavalletti M. Fornai F. Corsini G. U. (1996). Striatal MPP+ levels do not necessarily correlate with striatal dopamine levels after MPTP treatment in mice. Neurodegeneration 5, 129–136. 10.1006/NEUR.1996.0019 8819133

[B53] Wang Y.-J. Thomas P. Zhong J.-H. Bi F.-F. Kosaraju S. Pollard A. (2009). Consumption of grape seed extract prevents amyloid-β deposition and attenuates inflammation in brain of an Alzheimer’s disease mouse. Neurotox. Res. 15, 3–14. 10.1007/S12640-009-9000-X 19384583

[B54] Wang Y. Xu H. Fu Q. Ma R. Xiang J. (2011). Protective effect of resveratrol derived from Polygonum cuspidatum and its liposomal form on nigral cells in Parkinsonian rats. J. Neurol. Sci. 304 (1-2), 29–34. 10.1016/j.jns.2011.02.025 21376343

[B55] Zeng X.-S. Geng W.-S. Jia J.-J. (2018). Neurotoxin-induced animal models of Parkinson disease: pathogenic mechanism and assessment. ASN Neuro 10, 1759091418777438. 10.1177/1759091418777438 29809058 PMC5977437

[B56] Zhang Y. Huang N. Chen M. Jin H. Nie J. Shi J. (2019). Procyanidin protects against 6-hydroxydopamine-induced dopaminergic neuron damage via the regulation of the PI3K/Akt signalling pathway. Biomed. Pharmacother. 114, 108789. 10.1016/J.BIOPHA.2019.108789 30925459

[B57] Zhang Y. Lv C. Zhao G. (2021). Ways to enhance the bioavailability of polyphenols in the brain: A journey through the blood-brain barrier. Food Rev. Int. 1-17, 1–17. 10.1080/87559129.2021.1888973

