# Supplementary material for: Characterization of Nasco grape pomace-loaded nutriosomes and their neuroprotective effects in the MPTP mouse model of Parkinson’s disease
Source: Front Pharmacol. 2022 Aug 17;13:935784. doi: 10.3389/fphar.2022.935784 (PMC9428270; doi:10.3389/fphar.2022.935784)
Supplement: Supplementary file 1 [file DataSheet1.docx]

**Supplementary Data**

**Nutriosomes characterization**

The mean diameter, polydispersity index, and zeta potential of empty nutriosomes (EN) (control) and Nasco nutriosomes (NN) were measured (Table S2).

EN had a mean diameter ~188 nm and polydispersity index ~0.24, denoting a monodispersed system, and a zeta potential strongly negative, ~-59 mV (Table S2). The incorporation of the pomace extract at the lowest concentration (5 mg/ml) induced a slight, but significant, decrease in the vesicle size (~141 nm, Table S2). On the contrary, the incorporation of the highest concentration of pomace extract (10 mg/ml) led to an increase in vesicles' mean diameter, which was similar to that of empty ones, ~216 nm (Table S2). This modification of vesicle size is probably due to the intercalation of the lipophilic molecules contained in the extract in the lipid bilayer, causing a different bilayer assembling. The polydispersity index of NN decreased with respect to EN, being ~0.11 for NN (5 mg/ml) and ~0.19 for NN (10 mg/ml), indicating the prepared nutriosomes were homogeneous in size (Table S2). The zeta potential of NN was statistically equal to that of EN, confirming the loading of the extract inside the lipid bilayer. Of note, the zeta potential of NN (5 mg/ml) was less negative (~-65 mV) than that of NN (10 mg/ml) (~-49 mV) since the higher pomace dose vesicles were larger and delocalized the same charges within a larger surface area (Table S2). The physicochemical features of dispersions were measured over a period of 3-months upon storage at 25°C (Figure S1).


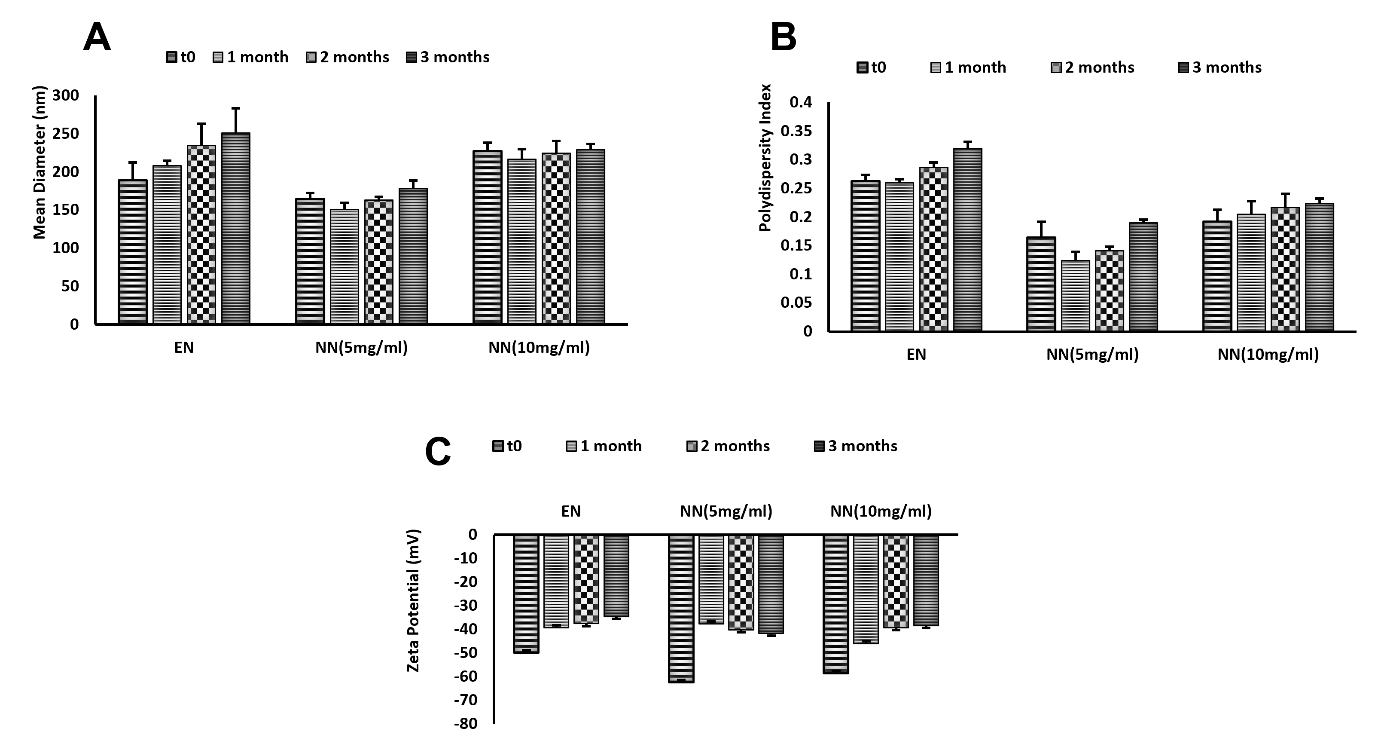


**Figure S1**. (A) Mean diameter, (B) polydispersity index, and (C) zeta potential of Nasco nutriosomes (NN, 5 and 10 mg/ml) stored for 3 months at 25°C. The data are represented as Mean ± SEM of at least six determinations.


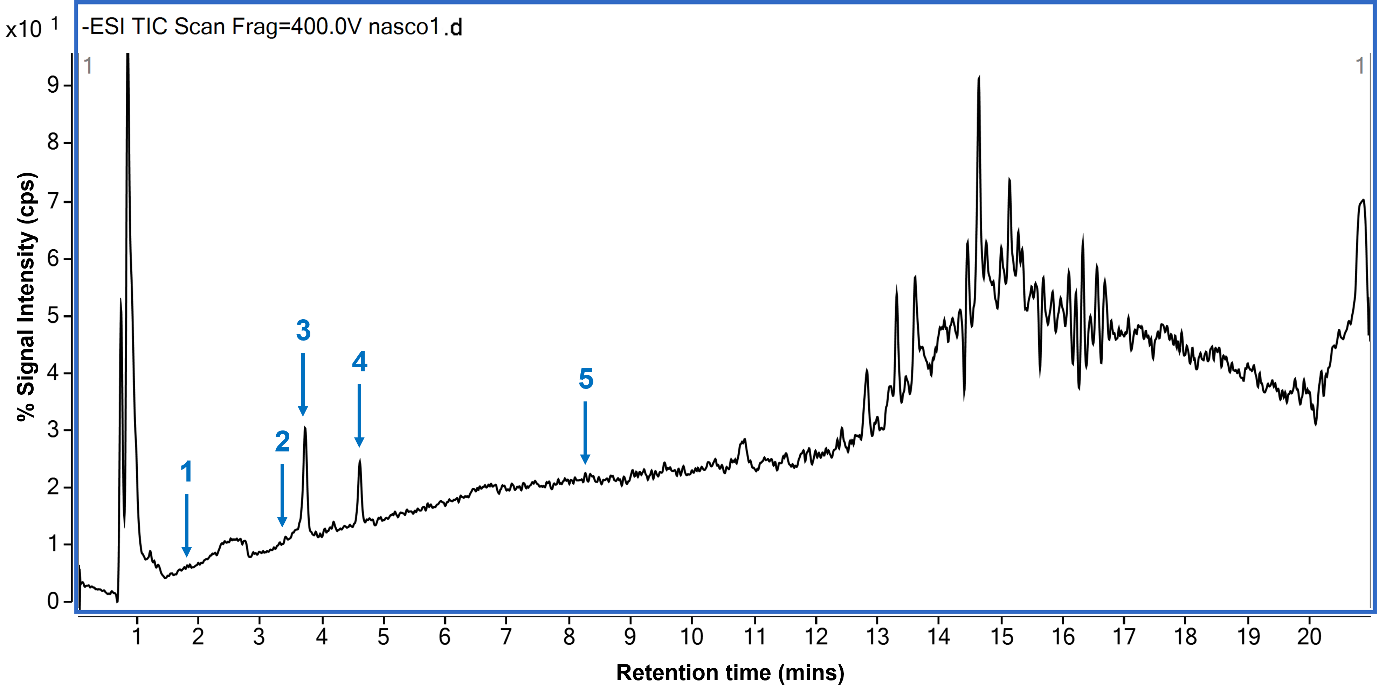


**Figure S2.** LC-QTOF-MS profiling of polyphenols present in NPE. Chromatogram refers to the total ion chromatogram (TIC) of extract obtained from Nasco grape pomace in the negative electrospray ionization (ESI-) mode. Peak assignation: (1) Gallic acid, (2) Procyanidin B2, (3) (+) Catechin, (4) (-) Epicatechin, and (5) Quercetin. Legend: X-axis represents retention time in minutes, Y-axis represents % signal intensity expressed as counts per second (cps). Abbreviation: NPE: Nasco pomace extract.


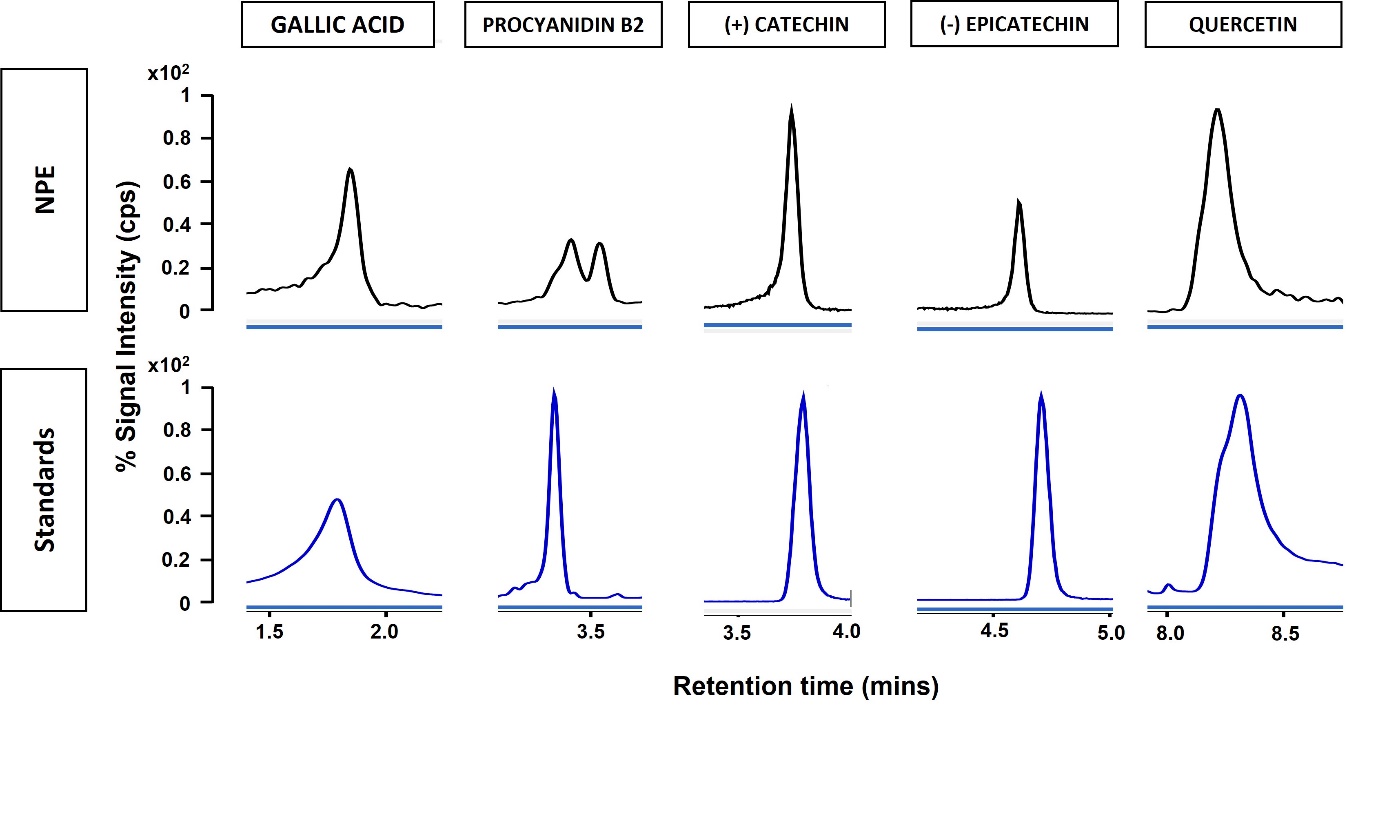


**Figure S3.** Comparison of Extracted Ion Chromatograms (EICs) of polyphenols from NPE and their respective analytical standards. Chromatograms of Gallic acid (169.0144, RT: 1.84), Procyanidin B2 (577.1351, RT: 3.41), (+) Catechin (289.0720, RT: 3.72), (-) Epicatechin (289.0719, RT: 4.60) and Quercetin (301.0360, RT: 8.21) obtained from NPE (top) and from analytical standards of respective compounds (bottom). Values in the bracket indicate the molecular mass of negatively charged molecular ion [M-H]^-^ and the retention time (RT), respectively. Legend: X-axis represents retention time in minutes, Y-axis represents % signal intensity expressed as counts per second (cps). Abbreviation: NPE: Nasco pomace extract.

**Table S1.** Composition of NN hydrated with a mixture of propylene glycol and water (50:50). Abbreviation: NN: Nasco nutriosomes.

|  | S75  (mg/ml) | Extract  (mg/ml) | Nutriose  (mg/ml) | Olive oil  (mg/ml) |
| --- | --- | --- | --- | --- |
| Empty-nutriosomes (EN) | 120 | 0 | 400 | 100 |
| Nasco nutriosomes (NN, 5 mg/ml) | 120 | 5 | 400 | 100 |
| Nasco nutriosomes (NN, 10 mg/ml) | 120 | 10 | 400 | 100 |

**Table S2.** Mean diameter (MD), polydispersity index (PI), zeta potential (ZP), and entrapment efficiency (EE) of empty nutriosomes (EN) and Nasco nutriosomes (NN). Every single value represents the average ± standard deviation of at least six determinations. The same symbol indicates the same value.

|  | MD  (nm) | PI | ZP  (mV) | EE  (%) |
| --- | --- | --- | --- | --- |
| Empty nutriosomes (EN) | °188±11 | 0.24±0.03 | ^+§^-59±5 | -- |
| Nasco nutriosomes  (NN, 5 mg/ml) | *141±15 | 0.11±0.02 | ^+^-65±2 | ^#^87±3 |
| Nasco nutriosomes  (NN, 10 mg/ml) | °216±26 | 0.19±0.04 | ^§^-49±9 | ^#^85±4 |

**Table S3.** **Quantitative analysis of polyphenols present in NPE.** The table represents the amounts of bioactive molecules (mg) contained in the extract (kg) obtained from Nasco grape pomace. The values are represented as Mean ± SEM (n=3). Δ ppm is the mean mass accuracy error. Abbreviation: NPE: Nasco pomace extract.

| Compounds in the NPE | Concentration level  (n=3; mg/kg) | Retention time (R_t_) (min) | Molecular Formula | Calculated Mass  [M-H]^-^ | Observed Mass  [M-H]^-^ | Δ ppm |
| --- | --- | --- | --- | --- | --- | --- |
| Hydroxybenzoic acid | | | | | | |
| Gallic acid | 182 ± 81 | 1.84 | C_7_H_6_O_5_ | 169.0142 | 169.0139 | 1.77 |
| Flavan-3-ols | | | | | | |
| (+) Catechin | 1375 ± 115 | 3.72 | C_15_H_14_O_6_ | 289.0710 | 289.0710 | 0 |
| (-) Epicatechin | 799 ± 67 | 4.60 | C_15_H_14_O_6_ | 289.0717 | 289.0717 | 0 |
| Procyanidin B2 | 4626 ± 46 | 3.41 | C_30_H_26_O_12_ | 577.1351 | 577.1344 | 1.21 |
| Flavonols | | | | | | |
| Quercetin | 1087 ± 60 | 8.21 | C_15_H_10_O_7_ | 301.0353 | 301.0337 | 5.31 |
